# Exploring Salivary Brain-Derived Neurotrophic Factor (BDNF) as a Potential Biomarker of Neuroplasticity in Older Adults Through Exergaming

**DOI:** 10.7759/cureus.93280

**Published:** 2025-09-26

**Authors:** Sarah C Pistritto, Amer M Burhan, Mary Chiu, Sara Elgazzar, Pritika Lally, Winnie Sun

**Affiliations:** 1 Faculty of Health Sciences, Ontario Tech University, Oshawa, CAN; 2 Department of Psychiatry, Ontario Shores Centre for Mental Health Sciences, Whitby, CAN; 3 Department of Psychiatry, Temerty Faculty of Medicine, University of Toronto, Toronto, CAN; 4 Department of Research and Academics, Ontario Shores Centre for Mental Health Sciences, Whitby, CAN; 5 Faculty of Science, Ontario Tech University, Oshawa, CAN

**Keywords:** brain-derived neurotrophic factor (bdnf), dna methylation, droplet digital pcr (ddpcr), exergaming, feasibility, late-life depression, persons with dementia, saliva collection

## Abstract

Background

Dementia and late-life depression (LLD) are common among older adults and are often associated with cognitive decline. Exergaming, which integrates physical and cognitive stimulation, may promote neuroplasticity in this population. Noninvasive biomarkers, such as salivary brain-derived neurotrophic factor (BDNF) methylation, provide a novel approach for monitoring intervention-related neuroplastic changes.

Objective

This pilot study examined the feasibility of a four-week exergaming intervention aimed at promoting both cognitive and physical engagement in older adults with dementia or LLD. The study also assessed changes in salivary BDNF DNA methylation, a biomarker of neuroplasticity, using noninvasive collection and droplet digital PCR (ddPCR) analysis.

Results

Participants engaged meaningfully in the exergame, and cognitive metrics showed improved performance across sessions. BDNF methylation was detectable in saliva samples, confirming feasibility; however, the small sample size and limited statistical power precluded significant findings. No causal conclusions can be drawn.

Conclusions

This study demonstrates that exergaming, combined with saliva collection and ddPCR, is both feasible and acceptable for older adults with cognitive impairment or depression. The intervention design was informed by theoretical frameworks of neuroplasticity, motor learning, and task-specific training. Larger controlled studies are warranted to evaluate clinical efficacy, expand BDNF analyses, and further investigate underlying neuroplastic mechanisms.

## Introduction

Dementia and late-life depression (LLD) are two highly prevalent conditions that affect cognitive function, emotional well-being, and quality of life in older adults. Dementia is characterized by significant cognitive decline across domains such as memory, language, attention, and executive function, which interferes with daily life [1]. LLD refers to a major depressive disorder occurring for the first time in individuals aged 60 or older. It shares overlapping neurobiological and clinical features with dementia, making differentiation difficult, as depression can both precede and coexist with cognitive decline, thereby increasing the risk of developing dementia [2]. As the aging population continues to grow, there is an urgent need for accessible, evidence-based, nonpharmacological interventions that can support brain health and mitigate functional decline in these populations [3].

Neuroplasticity is a fundamental mechanism supporting cognitive processes such as memory and learning [4]. However, accurately assessing, monitoring, and quantifying neuroplastic changes remains a challenge [5,6]. Physical activity (PA) and cognitive stimulation (CS) are well-established nonpharmacological approaches shown to improve cognitive function and mood in older adults with cognitive impairment and depressive symptoms [6]. In particular, planned, structured, and repetitive physical exercise may modulate brain structure and function by promoting the secretion of neurotrophic factors such as brain-derived neurotrophic factor (BDNF) [7,8]. BDNF supports neuronal growth, synaptic plasticity, and memory formation, processes essential for learning and memory [9]. Similarly, CS interventions have been shown to engage persons with dementia in activities that can enhance cognitive abilities [10,11]. These interventions aim to improve memory and attention, with activity difficulty adjusted as needed [12,13].

The role of exergaming

Exergaming is an interactive intervention that combines PA with cognitive engagement through gaming [14]. It has emerged as a novel, enjoyable, and accessible tool to support both physical and cognitive health. Activities such as aerobic exercise, dancing, balance training, and stretching promote user engagement while monitoring performance to assist in skill rehabilitation. A personalized approach allows participants to select functions tailored to their needs and preferences, enhancing uptake [15].

Despite its promise, only a small number of controlled studies have investigated whether exergaming can elicit a neuroplastic response [15]. To fully understand the potential of exergaming to promote neuroplasticity through CS and PA, further well-designed research is needed. Preliminary evidence suggests that integrating physical and cognitive tasks within an application may trigger neuroplastic responses mediated by BDNF secretion [8,16].

Salivary collection, DNA methylation, and digital PCR

Emerging research is exploring epigenetic mechanisms underlying behavioral change, including DNA methylation. DNA methylation is defined as the addition of a methyl group (-CH₃) that regulates gene expression by inhibiting the binding of transcription factors to DNA [17]. Regulation of DNA methylation is critical for normal cognitive function; altered methylation is associated with neurological disease and cognitive impairment. Methylation reflects cellular adaptability, as genes can be switched on or off depending on health status. In older adults, such alterations may contribute to Alzheimer’s disease or cognitive impairment.

Within DNA, CpG islands are often located near promoter regions, which regulate transcription. Methylation of CpG island promoters can reduce gene expression and impair transcription. DNA methylation is therefore critical for normal brain development and function, playing key roles in synaptic plasticity, neuronal repair, learning, and memory [18,19].

Salivary biomarkers, particularly BDNF, have emerged as promising indicators of neuroplasticity in populations with cognitive impairment. Saliva collection is noninvasive and simple, using either swabs or passive drool methods [20]. However, challenges remain in ensuring data quality, as saliva samples may contain bacterial DNA and show variability in cell type composition [21].

Digital PCR has been successfully applied to study DNA methylation in blood samples across age groups, including older adults with neurological conditions [22,23], though it has not yet been applied to saliva-based methylation analysis in this population. Digital PCR offers sensitivity and precision for detecting methylation changes in promoter regions and has been used to identify DNA methylation biomarkers for early cancer screening [24]. Thus, digital PCR holds promise for analyzing dementia-related biomarkers to measure, detect, and monitor disease progression.

Theoretical frameworks

The design of this study’s exergame intervention was guided by three frameworks. First, neuroplasticity refers to the brain’s ability to adapt in response to stimuli such as environmental changes, new learning experiences, or tasks [25]. A neuroplastic response is activated when the brain is stimulated through repetitive movements. Second, the motor learning framework proposes that new behaviors can be acquired through repeated practice and muscle memory [26]. Third, the task-specific training framework emphasizes the role of consistent repetition of specific activities or movements [27].

Together, these frameworks provide a foundation for an effective rehabilitative approach to optimize brain stimulation and modulate memory. The exergame intervention may act as a stimulus, promoting neuroplastic changes through repeated practice of both CS activities and PA movements. BDNF, a biomarker of learning- and memory-related neuronal activity, is naturally upregulated during exercise and can be noninvasively measured through saliva [28]. Based on this rationale, we hypothesize that regular participation in an integrated PA and CS exergame intervention will increase salivary BDNF levels, which may be positively associated with improvements in memory performance.

Study purpose

The purpose of this study was to explore the feasibility of a four-week exergaming intervention in older adults with dementia or LLD. The primary objective was to assess the feasibility and acceptability of the intervention in promoting cognitive and physical engagement. The secondary objective was to evaluate the feasibility of saliva collection and droplet digital PCR (ddPCR) analysis to detect changes in BDNF DNA methylation. This study was not designed to treat clinically diagnosed dementia or depression but to assess whether these approaches can be reliably implemented and measured in this population, providing preliminary insights for future research on accessible, personalized strategies to support cognitive health.

## Materials and methods

Study population and recruitment

This pilot feasibility study included five participants (one male and four females) with a formal diagnosis of either LLD or mild to moderate dementia (Table 1). All participants (n = 5) were screened for eligibility and met the inclusion criteria. One participant withdrew for personal reasons unrelated to the intervention; consequently, this individual’s salivary analysis was incomplete, and four samples were analyzed.

**Table 1:** Study demographics LLD, late-life depression; MMSE, Mini-Mental State Examination

Participant	MMSE score	Gender	Site	Diagnosis	Version of exergame chosen
P-11	24	M	Alzheimer’s Society (Alzheimer Society of Durham Region)	Cognitive impairment	Standing
P-34	29	F	Outpatient	LLD	Sitting
P-51	24	F	Outpatient	Cognitive impairment	Sitting
P-73	24	F	Outpatient	Cognitive impairment	Sitting
P-81	26	F	Outpatient	LLD	Sitting

Cognitive impairment was assessed using the Mini-Mental State Examination (MMSE) [29], administered by a research assistant to determine study eligibility. Individuals with MMSE scores between 12 and 25, indicative of mild to moderate impairment, were eligible for participation.

Additional inclusion criteria required participants to be aged 60 years or older and to be receiving care services from a local Alzheimer’s Society chapter or from an outpatient unit at a mental health hospital in Ontario, Canada. Exclusion criteria included a history of auditory or visual impairment, dry mouth or eyes, oral lesions, autoimmune diseases, electroconvulsive therapy within the past two weeks, difficulty communicating in English, or severe dementia (confirmed through medical diagnosis or MMSE). These criteria were designed to minimize potential bias and ensure reliable data collection, particularly regarding saliva collection and intervention effectiveness.

Exergaming sessions were conducted at the university and at the outpatient mental health hospital. Ethical approval for the study was obtained from the Research Ethics Boards of Ontario Shores Centre for Mental Health Sciences (JREB #22-033-B) and Ontario Tech University (REB #17059).

Research design 

Guided by three frameworks, such as (1) neuroplasticity, (2) motor learning, and (3) task-specific intervention, the exergame was designed to serve as a stimulus for participant engagement. BDNF is secreted during exercise and can be measured in saliva samples to detect whether learning is taking place. The innovative design of this exergame centers on the idea that engaging in specific physical exercises and cognitive challenges can stimulate the brain’s adaptive mechanisms to form new pathways that influence memory, mobility, and executive function. By incorporating these elements, the intervention aimed to maximize engagement and motivation, leading to improved health outcomes [15].

A secondary aim of the research design was to explore whether exergaming is a feasible method to engage participants in both PA and CS, with the potential to be tailored to individual needs to support cognitive health and overall well-being [28].

Data collection

Exergame Intervention 

This four-week pilot feasibility intervention involved twice-weekly sessions (~30 minutes each), for a total of eight sessions [30]. Each session alternated between a PA component (Zumba) and a CS task, with participants transitioning between the two until completion [30]. In total, participants completed five Zumba songs and four CS activities.

The exergame was custom-developed at Ontario Tech University for this study, rather than adapted from a commercial platform. It included interactive exercises specifically designed to promote cognitive and physical engagement in older adults [30]. Participant movements were tracked in real time, and accuracy was reflected through a star-based feedback system to provide motivation and encourage proper execution [30]. The exergame followed an aerobic protocol intended to stimulate BDNF secretion. Intensity varied by Zumba song, but movements were continuous and repetitive to induce aerobic exertion. Rest periods were provided during transitions to cognitive activities, reducing physical workload while maintaining mental engagement. This dosage protocol was guided by similar studies investigating exercise-based BDNF secretion [9,31,32]. Exercise intensity was not formally monitored via heart rate or exertion scales; participants performed activities at their preferred effort level.

CS tasks were programmed with standardized instructions, preset difficulty levels, and controlled timing to ensure consistent administration across participants [30]. Tasks were presented in a fixed order, and performance was tracked using metrics of accuracy, speed, and errors [30]. Visual feedback was provided in real time (e.g., matched cards remained face-up, puzzle pieces locked into place), and all actions and errors were logged automatically in a CSV file. Completion time was recorded to measure speed. Results were displayed in-game and exported as CSV files for review and analysis [30].

Saliva Sample Collection

Saliva samples were collected pre- and post-intervention using Saliva Self-Collection Kits (OG-600 model, DNA Genotek Inc., Ottawa, Canada) with the passive drool method (participants salivated through a funnel into a collection tube). Pre-intervention samples were collected before the first session, and post-intervention samples immediately after the eighth session. Participants were instructed to avoid drinking water for 15 minutes before the first pre-sample and to refrain from eating, smoking, or chewing gum for at least 30 minutes before each collection and until after the post-sample collection. Water was permitted after post-intervention collection. Samples were stored at room temperature until transported to The Centre for Applied Genomics (TCAG), Hospital for Sick Children, for analysis.

Saliva samples were analyzed at TCAG using ddPCR to assess DNA methylation at promoter IV sites (-39 and -35) of the BDNF gene. This site was selected because it is the most studied BDNF promoter region and is responsive to neuronal activity and synaptic plasticity. Prior literature also indicates methylation occurs within this promoter region [33]. The site contains CpG islands (5′CpG3′) that correlate with increased methylation, making this target suitable for ddPCR analysis and improving the likelihood of detecting methylation changes.

CS Activities

**Figure 1:**
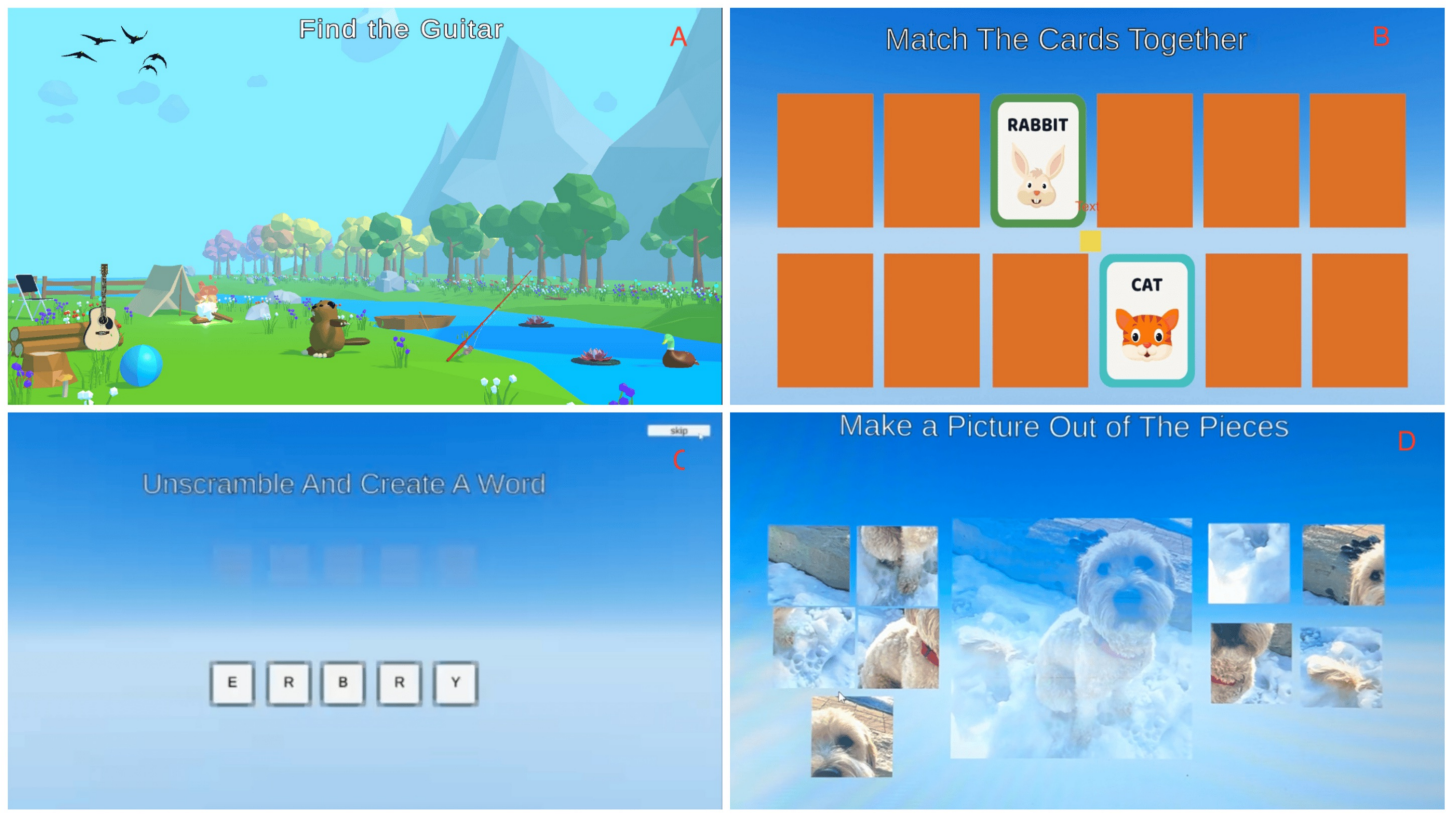
CS activities (A) Find Me. (B) Matching Cards. (C) Word Scramble. (D) Jigsaw Puzzle. CS, cognitive stimulation

Each of the four CS activities was carefully designed to stimulate the brain. The CS activity Find Me (Figure 1A) incorporates strategies aimed at enhancing cognitive abilities and optimizing learning processes. Through active reading, concentration, and the use of visual aids, this activity challenged participants’ comprehension and memory recall. It was intended to stimulate the frontal lobe (information processing) and the inferotemporal (IT) cortex (visual recognition).

The CS activity Matching Cards (Figure 1B) focuses on memory, attention, and concentration strategies to help keep the brain active and engaged. These strategies are aimed at potentially slowing cognitive decline and promoting short-term memory retention. This activity was designed to target the IT cortex for object recognition and visual pathways.

The CS activity Word Scramble (Figure 1C) engages the brain in problem-solving and linguistic tasks. The coordination of multiple brain areas during this activity can influence memory, attention, and executive function, thereby contributing to neuroplasticity. It was designed to promote cognitive flexibility (the ability to switch between tasks) and to stimulate Broca’s area (language) and the frontal lobe (working memory).

The CS activity Jigsaw Puzzle (Figure 1D) engages multiple cognitive processes, including memory, attention, and problem-solving. This activity was intended to stimulate the frontal lobe, particularly working memory.

Data analysis

Methylation Analysis of BDNF Promoter IV Site -39 and -35

Genomic DNA was extracted from saliva samples using the chemagicSTAR system (Hamilton Robotics, Reno, NV, USA). One microgram of gDNA from each sample was bisulfite-converted using the EpiTect Bisulfite Kit (Qiagen N.V., Hilden, Germany), following the manufacturer’s protocol. A ddPCR assay was designed against the bisulfite-converted sequence of the BDNF promoter IV (GRCh38 chr11:27,701,551-27,701,833) at sites -39 and -35 [33], using the primer and probe sequences listed in Table 2. The assay was ordered as a TaqMan Custom SNP Genotyping Assay (part 4332077; Thermo Fisher Scientific Inc., Waltham, MA, USA).

**Table 2:** Primer/probe and sequence

Primer or probe name	Sequence (5’ → 3’)
BSP forward	GGGTTGGAAGTGAAAATATTTGTAAAA
BSP reverse	CAAAAACAAAAAAATTTCATACTAATTC
Unmethylated (VIC) probe	TATTATATGATAGTGTATGTTAAGG
Methylated (FAM) probe	ATGATAGCGTACGTTAAG

Digital PCR was performed on the QX200 Droplet Digital PCR System (Bio-Rad Laboratories, Inc., Hercules, CA, USA). Each 20 µl reaction mixture contained 10 µl of 2× ddPCR SuperMix for Probes (Bio-Rad Laboratories), 0.5 µl of the 40× assay, 7.5 µl of water, and 50 ng of bisulfite-converted DNA (in a 2 µl volume). The assay was validated with a temperature gradient to ensure optimal separation of methylated (FAM) and unmethylated (VIC) signals. Positive control samples for methylated and unmethylated DNA were included (part D5014; Zymo Research Corp., Irvine, CA, USA).

Cycling conditions were as follows: 95°C for 10 minutes, followed by 45 cycles of 94°C for 30 seconds and 55°C for 1 minute, then 98°C for 10 minutes, and a final hold at 4°C on a Life Technologies Veriti Thermal Cycler. Data were analyzed using QuantaSoft Analysis Pro software v1.0.596 (Bio-Rad Laboratories).

Saliva Sample Statistical Analysis

Pre- and post-intervention data were examined using both descriptive and inferential statistics in SigmaPlot. Descriptive statistics, including visual droplet fluorescence amplitude plots generated by QuantaSoft Analysis Pro software v1.0.596, were used to summarize overall patterns in methylated (FAM) and unmethylated (VIC) sequences.

For exploratory purposes, simple linear regression analyses were performed to evaluate whether pre-intervention methylation values predicted post-intervention values for VIC (unmethylated) and FAM (methylated) measurements. Post-intervention values served as the dependent variable, and pre-intervention values as the independent variable. Paired t-tests were also conducted to compare pre- and post-intervention means. Model assumptions were assessed by examining the normality of residuals using the Shapiro-Wilk test and constant variance using the Spearman rank test. Model fit was evaluated using R² and adjusted R², while the significance of regression coefficients and overall models was assessed using t-tests and ANOVA, respectively. Statistical significance was defined as p < 0.05. Given the small sample size, these inferential analyses were underpowered and should be interpreted with caution. Accordingly, descriptive statistics were considered the primary method of representing saliva sample findings, with inferential results provided for preliminary insight.

CS Metrics

To measure participants’ cognitive performance across the eight sessions, embedded CS metrics, including speed, frequency of errors, and accuracy, were collected for each activity [30]. These data were analyzed descriptively pre- and post-intervention in SigmaPlot and presented in tables and graphs.

## Results

Saliva samples

Four participants completed pre- and post-intervention saliva samples, from which DNA of the BDNF gene was extracted and analyzed for methylation using two probes (VIC, unmethylated; and FAM, methylated). DNA extraction and digital PCR analysis were conducted and analyzed by a third party at the SickKids Centre for Applied Genomics Laboratory.

**Figure 2:**
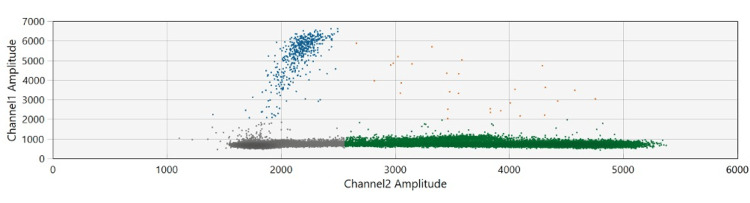
Fluorescence amplitude plot Channel 1: FAM methylated, represented by blue dots. Channel 2: VIC unmethylated, represented by green dots. The grey cluster represents empty droplets, whereas the orange dots represent a mixture of both methylated and unmethylated droplets.

Figure 2 presents the fluorescence amplitude droplets of the two probes: Channel 1 (FAM, methylated) is represented by blue dots, and Channel 2 (VIC, unmethylated) by green dots. The grey cluster represents empty droplets, which is expected in this type of experiment. The orange dots represent droplets containing both methylated and unmethylated sequences. This plot demonstrates that the probes effectively detected a small amount of methylated sequence at this promoter site, as shown by the distinct cluster of blue dots. This finding suggests that a neuroplastic response may have been activated when the brain was repeatedly stimulated, either through ZUMBA PA or CS tasks.

Inferential statistics results

A pre- and post-intervention linear regression and paired t-test were conducted with four participants. Participant P-73 dropped out after the third session; therefore, her post-intervention saliva collection was incomplete.

Regression Analysis

For FAM (methylated), post-intervention values were moderately predicted by pre-intervention values (Post = 11.808 + 0.575 × Pre; R = 0.804, R² = 0.646). However, the model was not statistically significant (p = 0.196) and demonstrated low statistical power (0.198 vs. desired 0.800), indicating that the findings should be interpreted with caution. For VIC (unmethylated), regression revealed only a weak relationship between pre- and post-intervention levels (R² = 0.158, p = 0.602), with insufficient power (0.062 vs. 0.800), further reinforcing the need for cautious interpretation (Table 3, Table 4).

**Table 3:** VIC unmethylated linear regression Linear regression of post- versus pre-intervention values (N = 4, 1 missing) showed nonsignificant coefficients (constant p = 0.145; pre p = 0.602) and low explanatory power (R² = 0.158). The ANOVA was also nonsignificant (F = 0.376, p = 0.602). Residuals were normally distributed (p = 0.962), but variance was unequal (p < 0.001). Low statistical power (0.062) indicates the need for cautious interpretation (p < 0.05 considered significant).

Parameter/section	Value/coefficient	Standard error	t	F	p
Analysis date/time	February 19, 2024 1:01:42 AM	N/A	N/A	N/A	N/A
Data source	VIC unmethylated (pre/post)	N/A	N/A	N/A	N/A
Regression equation	Post = 6756.741 + 0.218 × Pre	N/A	N/A	N/A	N/A
N	4	N/A	N/A	N/A	N/A
Missing observations	1	N/A	N/A	N/A	N/A
R	0.398	N/A	N/A	N/A	N/A
R²	0.158	N/A	N/A	N/A	N/A
Adjusted R²	0	N/A	N/A	N/A	N/A
Standard error of estimate	1767.22	N/A	N/A	N/A	N/A
Coefficient: Constant	6756.741	2897.144	2.332	N/A	0.145
Coefficient: Pre-intervention	0.218	0.356	0.613	N/A	0.602
ANOVA: Regression	1	1174757	1174757	0.376	0.602
ANOVA: Residual	2	6246134	3123067	N/A	N/A
ANOVA: Total	3	7420891	2473630	N/A	N/A
Normality (Shapiro-Wilk)	Passed	N/A	N/A	N/A	0.962
Constant variance (Spearman rank)	Failed	N/A	N/A	N/A	<0.001
Power (α = 0.05)	0.062	N/A	N/A	N/A	N/A

**Table 4:** FAM methylated linear regression Linear regression predicting post-intervention FAM methylation from pre-intervention values (N = 4, 1 missing) was not statistically significant (p > 0.05). Residuals were normally distributed (p = 0.556), but variance was unequal (p < 0.001). Low power (0.198) further limits interpretation.

Parameter/section	Value/coefficient	Standard error	t	F	p
Analysis date/time	February 19, 2024 12:54:21 AM	N/A	N/A	N/A	N/A
Data source	FAM methylated pre/post	N/A	N/A	N/A	N/A
Regression equation	Post 8-session intervention = 11.808 + 0.575 × Pre-intervention	N/A	N/A	N/A	N/A
N	4	N/A	N/A	N/A	N/A
Missing observations	1	N/A	N/A	N/A	N/A
R	0.804	N/A	N/A	N/A	N/A
R²	0.646	N/A	N/A	N/A	N/A
Adjusted R²	0.47	N/A	N/A	N/A	N/A
Standard error of estimate	4.768	N/A	N/A	N/A	N/A
Coefficient: Constant	11.808	4.663	2.532	N/A	0.127
Coefficient: Pre-intervention	0.575	0.301	1.912	N/A	0.196
ANOVA: Regression	1	83.077	83.077	3.655	0.196
ANOVA: Residual	2	45.458	22.729	N/A	N/A
ANOVA: Total	3	128.535	42.845	N/A	N/A
Normality (Shapiro-Wilk)	Passed	N/A	N/A	N/A	0.556
Constant variance (Spearman rank)	Failed	N/A	N/A	N/A	<0.001
Power (α = 0.05)	0.198	N/A	N/A	N/A	N/A

Paired t-Tests

**Figure 3:**
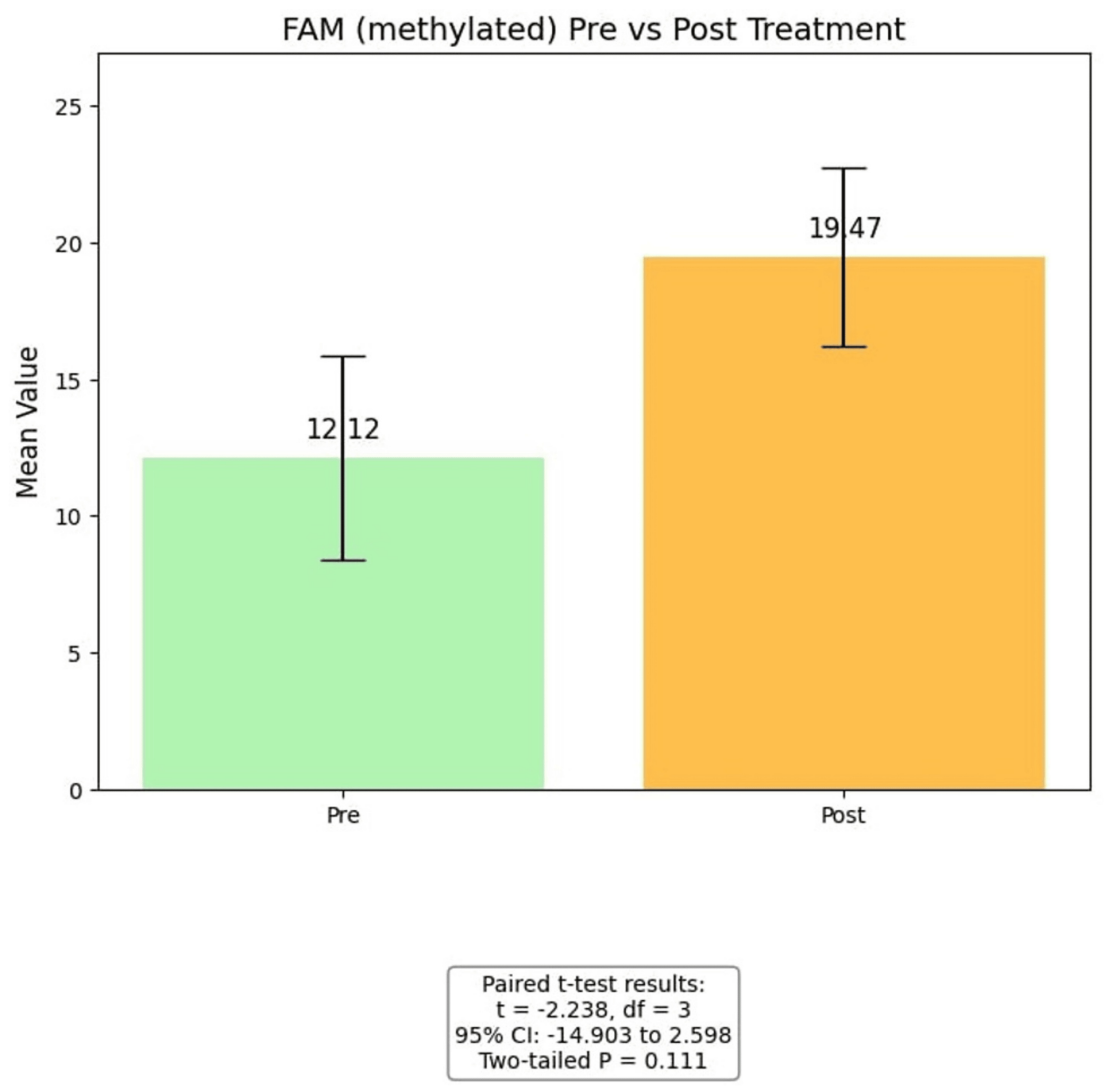
t-Test FAM (methylated) Pre- and post-treatment levels of FAM (methylated) in the sample (n = 5). Bars represent mean values with error bars indicating the standard error of the mean (SEM). A paired t-test was conducted to assess differences between pre- and post-treatment values. The mean difference was -6.15, with t(3) = -2.238, 95% CI (-14.90, 2.60), and a two-tailed p = 0.111, indicating that the observed change was not statistically significant.

**Figure 4:**
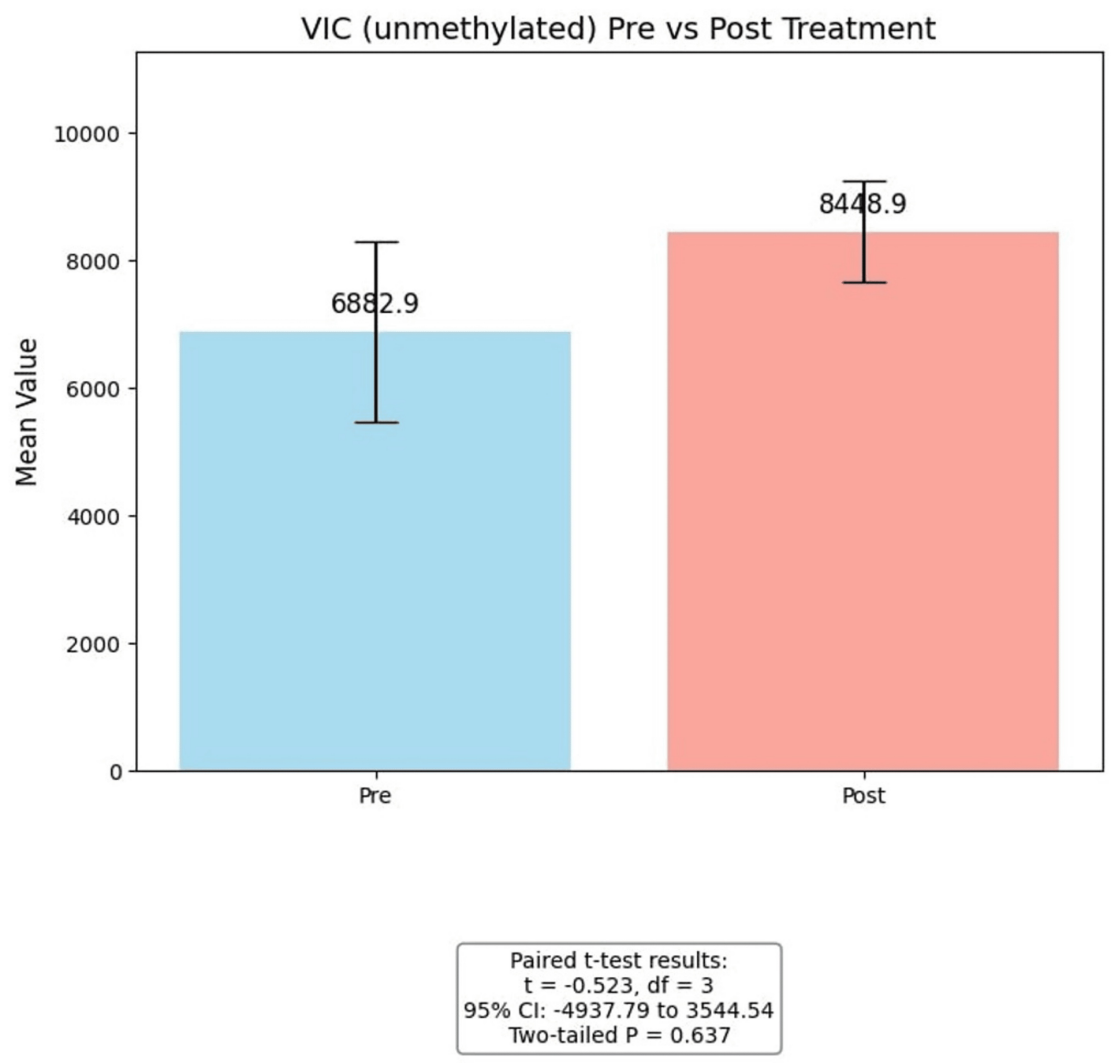
t-Test VIC (unmethylated) Pre- and post-treatment levels of VIC (unmethylated) in the sample (n = 5). Bars represent mean values with error bars indicating SEM. A paired t-test was conducted to assess differences between pre- and post-treatment values. The mean difference was -696.63, with t(3) = -0.523, 95% CI (-4937.79, 3544.54), and a two-tailed p = 0.637, indicating that the observed change was not statistically significant.

For FAM (methylated), post-intervention values (19.47 ± 6.55) were higher than pre-intervention values (12.12 ± 8.36). While the one-tailed test approached significance (p = 0.0556), the two-tailed test did not (p = 0.111), suggesting a trend toward increased methylation following the intervention, though limited by sample size. For VIC (unmethylated), post-intervention values (8448.94 ± 1572.78) were slightly higher than pre-intervention values (6882.86 ± 3153.34), but the difference was not statistically significant (t = -0.523, p = 0.637). Overall, these results suggest a possible increase in methylation-specific signals post-intervention, though the small sample size and low power limit definitive conclusions (Figure 3, Figure 4).

CS results

Find Me

**Figure 5:**
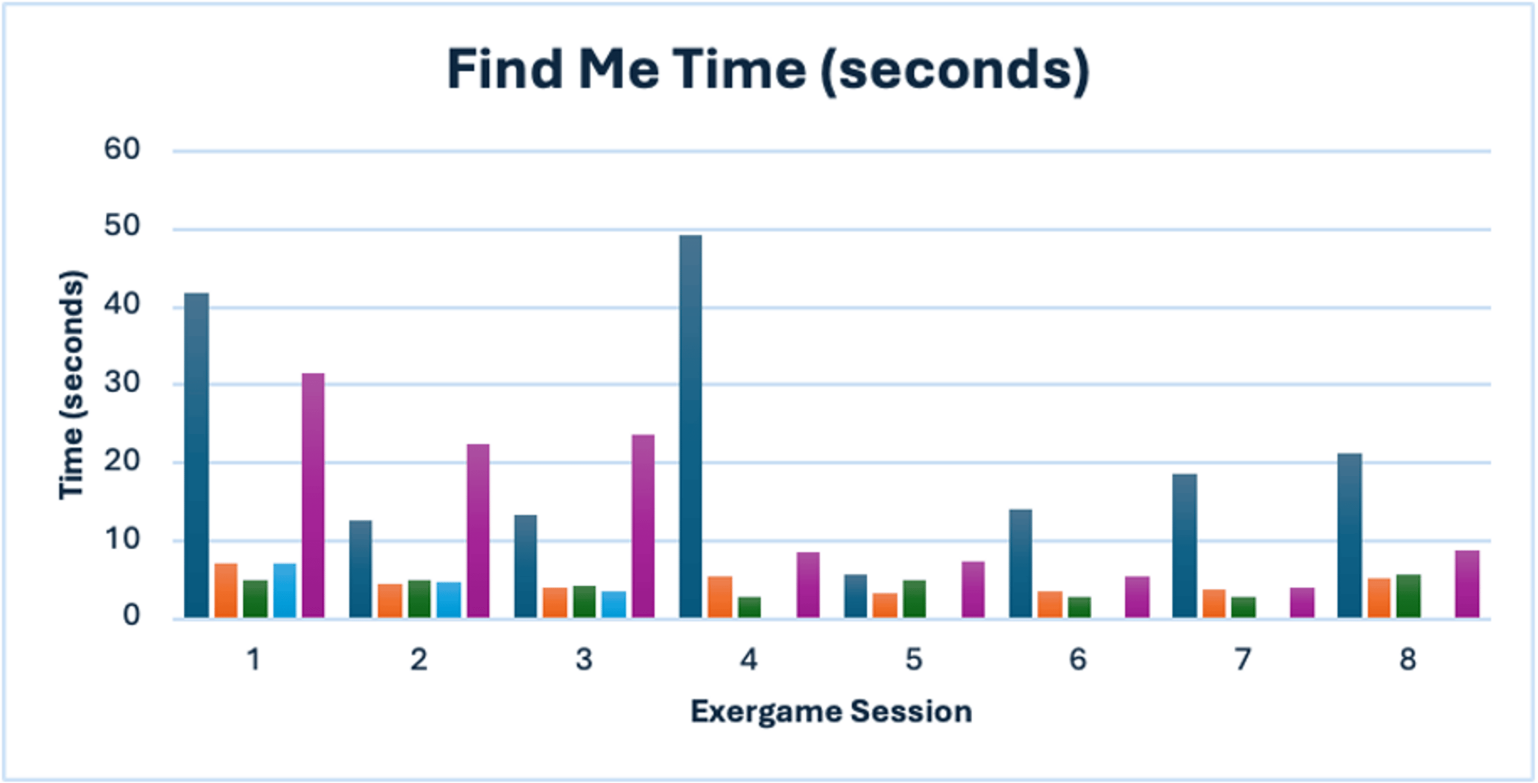
Find Me time (seconds) Navy blue = P-11; Orange = P-34; Green = P-51; Blue = P-73; Purple = P-81

This activity tracked improvements in speed and frequency of error (corresponding to instances where participants identified the wrong object) pre- and post-intervention. Participant P-73 dropped out after the third session; therefore, her post-scores were missing (Table 5, Figure 5).

**Table 5:** Find Me errors Number of Find Me errors observed for each participant across sessions S1-S8. Values indicate the count of errors per session. “0” indicates no errors. “N/A” indicates that the item was not applicable or not assessed for that participant.

Session	FIND ME errors - Group				
	P-11	P-34	P-51	P-73	P-81
S1	1	0	0	0	0
S2	0	0	1	1	1
S3	1	0	0	0	2
S4	0	0	0	N/A	0
S5	0	0	0	N/A	0
S6	0	0	0	N/A	0
S7	0	0	0	N/A	0
S8	0	0	0	N/A	0

Matching Cards

**Figure 6:**
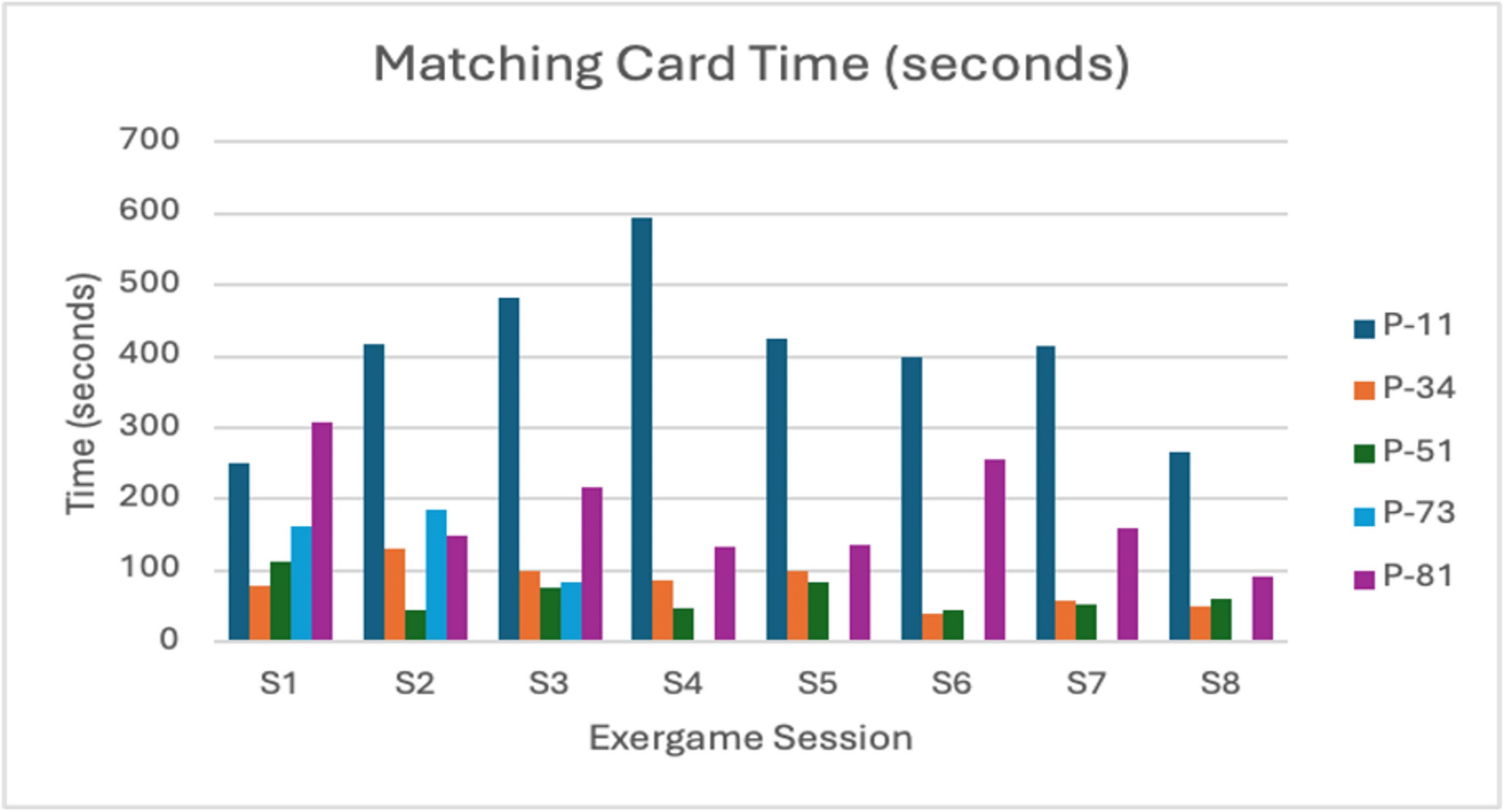
Matching Card time (seconds) Navy blue = P-11; Orange = P-34; Green = P-51; Blue = P-73; Purple = P-81

**Figure 7:**
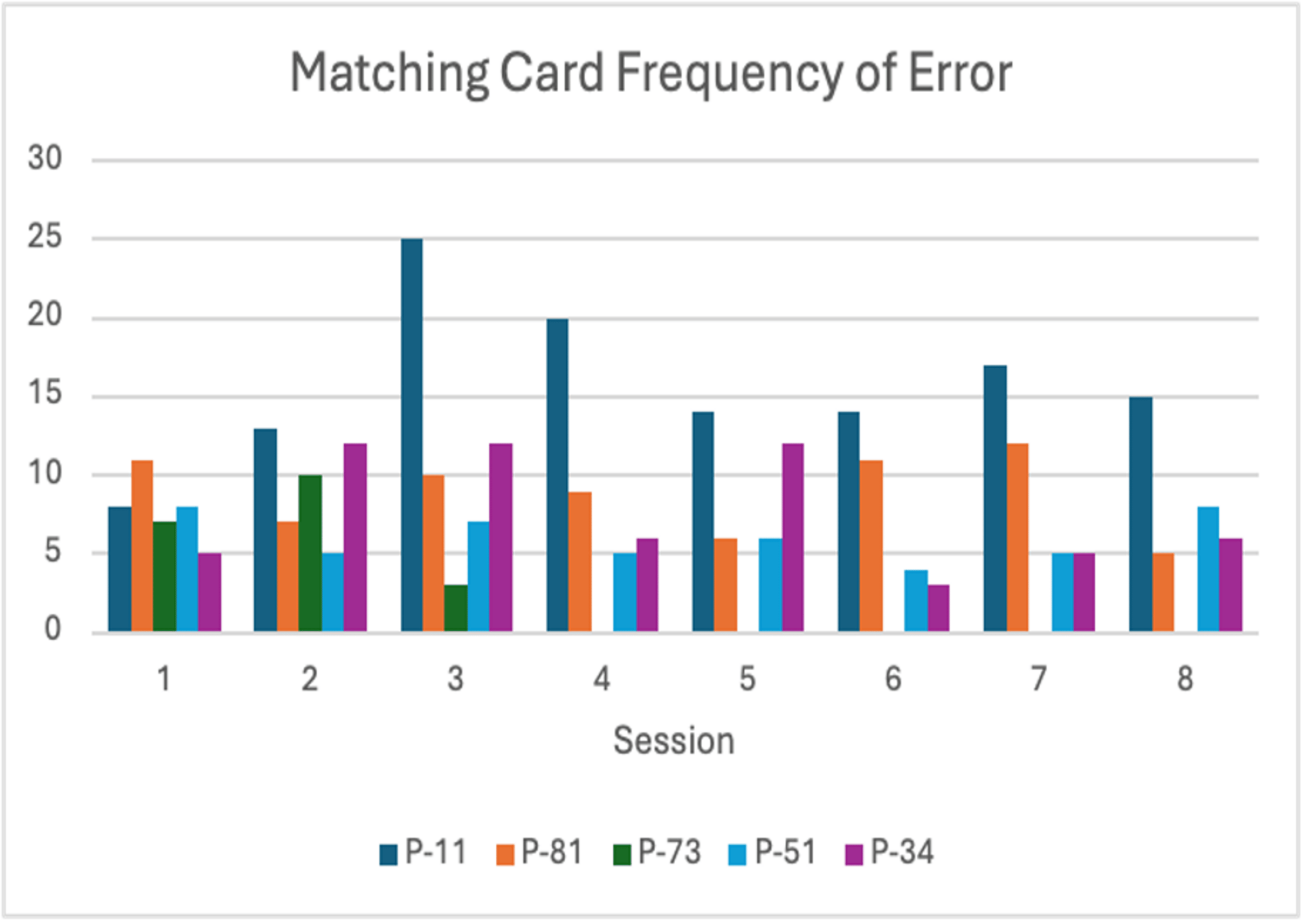
Matching Card errors Navy blue = P-11; Orange = P-34; Green = P-51; Blue = P-73; Purple = P-81

This activity tracked speed improvements pre- and post-intervention, as well as frequency of errors across sessions. Participant P-73 dropped out after the third session; therefore, her post-scores and subsequent session data are incomplete (Figure 6, Figure 7).

Word Scramble 

**Figure 8:**
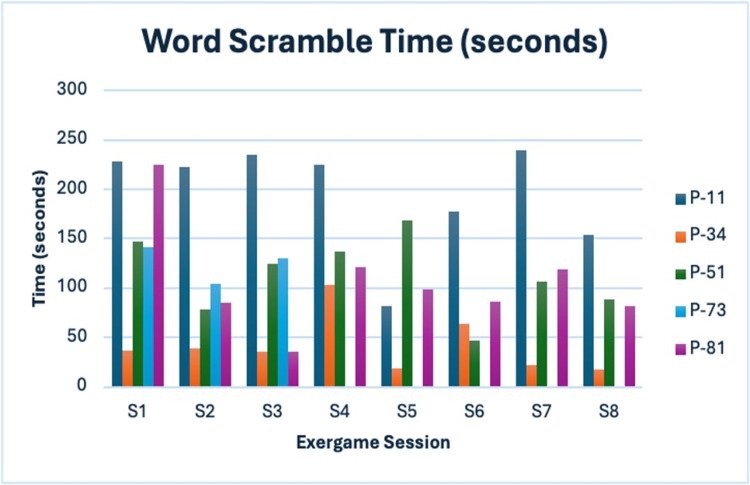
Word Scramble time (seconds) Navy blue = P-11; Orange = P-34; Green = P-51; Blue = P-73; Purple = P-81

This activity tracked speed improvements pre- and post-intervention. Participant P-73 dropped out after the third session; therefore, her post-scores and subsequent session data are incomplete (Figure 8).

Jigsaw Puzzle

**Figure 9:**
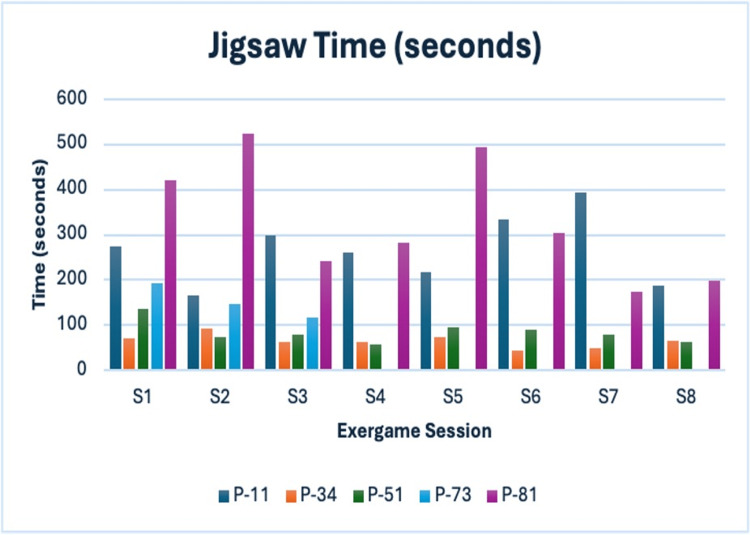
Jigsaw time (seconds) Navy blue = P-11; Orange = P-34; Green = P-51; Blue = P-73; Purple = P-81

This activity tracked speed improvements pre- and post-intervention. Participant P-73 dropped out after the third session; therefore, her post-scores and subsequent session data are incomplete (Figure 9).

## Discussion

This study demonstrates several key strengths. It presents an innovative integration of exergaming with noninvasive saliva collection and ddPCR analysis of the BDNF gene at promoter IV sites -39 and -35, offering a novel approach to examine neuroplasticity biomarkers in older adults with dementia or LLD. The molecular methods are rigorously described, enhancing methodological transparency, and the intervention is grounded in a strong theoretical framework that combines cognitive and physical stimulation. Despite the small sample size, the study successfully demonstrated the feasibility of implementing the exergaming intervention and collecting saliva samples, providing valuable insight for future research. Additionally, real-time performance tracking and structured cognitive tasks offer preliminary evidence that these methods can be applied to monitor behavioral and cognitive engagement in this population.

Engagement in exergame

Participants engaged meaningfully in the exergame intervention, and the study demonstrated the integration of quantitative exergame CS metrics with salivary BDNF methylation data to explore neuroplasticity. Exergaming provided a motivating way to combine PA with cognitive tasks, potentially influencing learning and memory processes. This was evident in participants’ cognitive performance metrics and corresponding BDNF methylation results. Qualitative thematic analysis from Pistritto et al. [30] also indicated that the program positively influenced cognitive, physical, and psychosocial health domains. These findings highlight the potential for exergaming not only as a therapeutic tool but also as a research platform for investigating neuroplasticity and epigenetic biomarkers in neuropsychiatric populations. This pilot study demonstrates that saliva collection and ddPCR are feasible and reliable in this population, and that exergaming is both acceptable and engaging. While preliminary data suggest potential changes in BDNF methylation, these findings were not statistically significant and cannot establish causality. Future large-scale, controlled trials are required to validate these results and explore clinical applications.

BDNF methodology and feasibility collection

Traditionally, BDNF has been measured through blood samples because it is produced in the brain and released into the bloodstream. However, this study adopted a novel, noninvasive approach by measuring BDNF through saliva samples, a method that is relatively uncommon [32]. This approach offers increased accessibility and is less invasive for older adults, potentially improving participant compliance [34].

Secondly, the use of ddPCR to detect BDNF methylation in saliva has not previously been applied in this context. Other researchers have used techniques such as ELISA [35] and MassARRAY [36], as confirmed by lab experts from SickKids Hospital Centre for Applied Genomics.

Thirdly, the focus on BDNF promoter IV CpG sites -39 and -35 was informed by guidance from experts at SickKids Hospital and findings reported by Pathak et al. [33], who demonstrated that methylation at CpG-39 significantly impacted promoter activity (under antidepressant treatment conditions). To align with the cAMP response element (CRE) region highlighted in Pathak et al., our assay design was modified to target this area, with the goal of improving base composition compatibility for the ddPCR environment. This region has not been well characterized in prior research on DNA methylation and neuroplasticity in dementia populations, positioning our work as a novel contribution that may provide new insights into epigenetic mechanisms relevant to dementia and LLD care.

Saliva samples were collected using DNA Genotek kits, recommended by an expert advisor from the Centre for Addiction and Mental Health, which have been clinically validated in previous DNA methylation studies [35,37-39]. Together, these methodological decisions contributed to a more accessible, precise, and scalable framework for future studies investigating the epigenetic underpinnings of neuroplasticity in aging populations.

Methodologically, the fluorescence amplitude plot from the ddPCR assay visually indicated the presence of BDNF methylation at this promoter region. However, the extent of methylation could not be reliably quantified due to the small sample size and limited statistical power. Similar observations were reported by Pathak et al. [33] in studies involving antidepressants. The detection of methylation at this site supports the feasibility of saliva collection and demonstrates that ddPCR is a reliable method for this type of analysis. While preliminary data suggest potential BDNF methylation changes, the findings were not statistically significant and cannot establish causality. Future large-scale, controlled trials are needed before clinical applications can be considered.

Theoretical frameworks

Study findings suggested that the three guiding frameworks, neuroplasticity, motor learning, and task-specific training, embodied within the exergame program, were a feasible approach to facilitate participatory exergame design. A neuroplastic response appeared to be activated when the brain was continually stimulated through either PA or cognitive tasks. Each time a skill or activity is performed, the brain refines and reinforces the motor pathway, regardless of execution accuracy [40].

This was evident in the data from the Matching Cards activity, where response times decreased while error frequency also declined, suggesting that participants improved in accuracy and, consequently, speed. Observations by the research team also noted that participants’ interaction with the ZUMBA PA avatar improved, as they mirrored the avatar’s movements more quickly over time. These findings suggest that participants’ psychomotor speed, memory recall, cognition, and mobility improved with continued engagement, likely due to practice and task repetition.

Our findings regarding psychomotor speed align with results from Karssemeijer et al. [41], who conducted a randomized controlled trial in persons with dementia and reported improved psychomotor speed following a 12-week exergaming program. This was reflected in some of our participants’ results, particularly after sessions three and four. As psychomotor speed typically declines with age, pilot data from our study suggest that integrated PA and CS through exergaming could be a useful tool to enhance psychomotor speed and cognitive function.

Limitations

Participant recruitment posed a challenge for both study populations (persons with dementia and individuals with LLD), requiring nearly six months to recruit and enroll participants and resulting in a small sample size. Findings are therefore preliminary and not generalizable. Although inferential statistics were limited by the small sample size, the findings provide initial evidence supporting the feasibility of assessing DNA methylation changes in BDNF, with descriptive statistics offering the most reliable representation of the results.

Another limitation is that this study assessed only the BDNF promoter IV sites -39 and -35. Other BDNF gene regions may exhibit higher levels of methylation. Additionally, this study did not assess BDNF gene expression in specific diseases such as Alzheimer’s disease or cognitive impairment. Investigating these associations would require isolating RNA and performing next-generation sequencing of the entire BDNF gene. Although this approach is more comprehensive and costly, it could provide more targeted information on methylation patterns and potential predictive biomarkers.

Saliva sample variability, including differences in cell composition, was not assessed and could influence DNA methylation measurements. Furthermore, only basic demographic information (gender and age ≥60 years) was collected. Additional clinical and demographic data were not gathered, limiting the ability to explore how these factors might influence the relationship between LLD and dementia.

## Conclusions

This pilot study demonstrates the feasibility of combining exergaming with noninvasive saliva collection and ddPCR to examine BDNF promoter IV methylation in older adults with dementia or LLD. The intervention was grounded in theoretical frameworks of neuroplasticity, motor learning, and task-specific training, which guided the design of integrated cognitive and physical activities. Participants engaged meaningfully, and real-time cognitive performance metrics suggest that this approach can monitor behavioral and cognitive engagement. While BDNF methylation was detectable, the small sample size and limited power prevented reliable quantification or causal conclusions. Limitations include analysis restricted to promoter IV sites, unassessed saliva cell composition, and minimal demographic and clinical data. Overall, this study provides proof-of-concept evidence supporting feasibility and highlights the potential for future research to optimize exergame design, expand BDNF methylation analysis, and examine long-term clinical impacts.
